# Trace Elements Status and Metallothioneins DNA Methylation Influence Human Hepatocellular Carcinoma Survival Rate

**DOI:** 10.3389/fonc.2020.596040

**Published:** 2021-01-28

**Authors:** Silvia Udali, Domenica De Santis, Filippo Mazzi, Sara Moruzzi, Andrea Ruzzenente, Annalisa Castagna, Patrizia Pattini, Greta Beschin, Antonia Franceschi, Alfredo Guglielmi, Nicola Martinelli, Francesca Pizzolo, Francesca Ambrosani, Oliviero Olivieri, Sang-Woon Choi, Simonetta Friso

**Affiliations:** ^1^ Department of Medicine, University of Verona, Verona, Italy; ^2^ Department of Surgery, University of Verona, Verona, Italy; ^3^ Department of Diagnostics and Public Health, University of Verona School of Medicine, Verona, Italy; ^4^ Chaum Life Center, CHA University School of Medicine, Seoul, South Korea; ^5^ Department of Nutrition, University of Massachusetts School of Public Health and Health Sciences, Amherst, MA, United States

**Keywords:** copper, DNA methylation, epigenetics, hepatocellular carcinoma, *MT1G*, *MT1H*, survival rate, trace elements

## Abstract

**Background:**

Mechanisms underlying hepatocellular carcinoma (HCC) development are largely unknown. The role of trace elements and proteins regulating metal ions homeostasis, i.e. metallothioneins (MTs), recently gained an increased interest. Object of the study was to investigate the role of promoter DNA methylation in MTs transcriptional regulation and the possible prognostic significance of serum trace elements in HCC.

**Methods:**

Forty-nine HCC patients were enrolled and clinically characterized. Cu, Se, and Zn contents were measured by Inductively Coupled Plasma Mass Spectrometry in the serum and, for a subset of 27 patients, in HCC and homologous non-neoplastic liver (N) tissues. *MT1G* and *MT1H* gene expression in hepatic tissues was assessed by Real-Time RT-PCR and the specific promoter DNA methylation by Bisulfite-Amplicon Sequencing.

**Results:**

Patients with Cu serum concentration above the 80^th^ percentile had a significantly decreased survival rate (P < 0.001) with a marked increased hazard ratio for mortality (HR 6.88 with 95% CI 2.60–18.23, P < 0.001). Se and Zn levels were significantly lower in HCC as compared to N tissues (P < 0.0001). *MT1G* and *MT1H* gene expression was significantly down-regulated in HCC as compared to N tissues (P < 0.05). MTs promoter was hypermethylated in 9 out of the 19 HCC tissues showing MTs down-regulation and methylation levels of three specific CpGs paralleled to an increased mortality rate among the 23 patients analyzed (P = 0.015).

**Conclusions:**

*MT1G* and *MT1H* act as potential tumor suppressor genes regulated through promoter DNA methylation and, together with serum Cu concentrations, be related to survival rate in HCC.

## Introduction

Liver cancer is the sixth most commonly diagnosed cancer and the fourth leading cause of cancer death worldwide, and hepatocellular carcinoma (HCC) is the most frequent primary liver cancer (1). In the majority of cases, HCC develops within an established background of chronic liver disease (70–90% of all patients) caused by hepatitis C virus (HCV) or hepatitis B virus (HBV) infection or by alcohol abuse (2). However, the etiology remains largely unknown, especially for the non-viral HCC and the interest to identify circulating markers of survival is object of experimental research. Particular attention was recently dedicated to the role of specific trace elements in liver carcinogenesis. In addition to iron, whose overload is associated to the production of free oxygen radicals and subsequent damage of nucleic acid as the possible trigger for hepatocarcinogenesis (3), Cu, Se, and Zn have been also evoked in association with primary liver cancers development (4, 5). Copper participates to the production of oxygen free radicals and its accumulation in the liver seems the most plausible trigger toward the carcinogenetic processes (6). Selenium is involved in the protection against free radicals, being an essential component of the antioxidative glutathione peroxidases pathway, and Se deficiency was reported in association with increased incidence of HCC (7, 8). Zinc is an essential metal for life that mainly acts as an antioxidant, even though, in the case of intracellular Zn overload it can also be involved, on the contrary, in promoting cellular oxidative stress (9). The role of Zn, however, has been mostly studied in relation to its antioxidant activity through the catalytic action of copper/zinc-superoxide dismutase, the protection of the protein sulfhydryl groups, and the up-regulation of metallothioneins (MTs). MTs are low molecular weight cysteine-rich intracellular proteins capable to bind essential and toxic metals. They lead a pivotal role in the cellular functions of detoxification and protection against oxidative stress, due to their ability to scavenge free radicals (10). Recently, MT1 isoforms, in particular MT1M and MT1G, were suggested as possible biomarkers for HCC and other human cancers (11). A critical role in cancer development is also played by DNA methylation, the epigenetic phenomenon that consists in the addition of methyl groups to cytosines within CpG dinucleotide sequences and affects gene expression and genomic stability (12–14). An aberrant DNA methylation, in particular gene-specific hypermethylation and genomic DNA hypo-methylation, has been observed in many cancer tissues and cancer precursor cells (15) suggesting DNA methylation as a suitable cancer biomarker (16). In a previous study, we characterized genome-wide DNA methylation and gene expression profiles of HCC tissues by microarray technologies and observed that promoter hypermethylation is associated to transcriptional repression of two MTs, suggesting a role for DNA methylation in gene silencing (17). The first aim of the present study was to investigate the role of trace elements in HCC by analyzing their content in serum and liver tissues and consequently to evaluate a possible correlation between serum trace elements concentrations and survival rate. Additional purposes were to unravel the function of promoter DNA methylation in regulating *MT1G* and *MT1H* gene expression and to assess the possible correlation of MTs promoter methylation with HCC survival rate.

## Materials and Methods

### Study Subjects: Biochemical Analysis, Clinical History Data, and Biological Samples Collection

Forty-nine patients affected by HCC were enrolled from those referring to the Division of Hepatobiliary Surgery of the Verona University Hospital (Verona, Italy) for curative surgery intervention. Detailed enrolment criteria were previously reported (18), in particular exclusion criteria were coexisting HBV or HCV infections and surgical resectability criteria were a preserved liver function, Child- Pugh class A, the presence of a resectable single tumor or oligofocal resectable nodules (maximum three nodules), and the absence of extrahepatic metastases. The resectability assessment also included the tumor local stage, major vascular invasion, and the presence of affected lymphonodes (17, 18). The study protocol conformed to the ethical guidelines of the 1975 Declaration of Helsinki and was approved by the Ethical Review Board of the University of Verona School of Medicine Hospital (Verona, Italy). Written informed consent was obtained from each patient after a detailed explanation of the study. Routine laboratory tests were performed including a complete blood count, indexes of hepatic function, and serological tests for HBV and HCV. A detailed clinical history was recorded for all the patients and a periodic evaluation was performed during a follow-up period up to 110 months. Data of tumor grading and microvascular invasion were available for 40 out of 49 subjects (81.6%). The follow-up period was calculated from the date of surgery until the last date of follow-up visit or to the date of death.

### Serum and Tissue Samples Collection

Before the surgical intervention, blood samples were drawn from each patient in BD Vacutainer**^®^** tubes without anticoagulant reagents, centrifuged at 2.500 × g for 15 min at room temperature and the serum was collected and stored at −20°C for subsequent analyses. HCC and homologous non-neoplastic liver (N) tissues were collected immediately after surgical excision, sliced into aliquots of about 100 mg, snap-frozen in liquid nitrogen, and stored at −80°C. Aliquots for RNA extraction were immediately homogenized in 2 ml of TRI**^®^** Reagent (Sigma-Aldrich, St. Louis, MO, USA) and the homogenate stored at −80°C until use. A pathologist unaware of the patient participation to the study performed the histological analysis of liver tissues.

### Trace Elements Analysis in Serum and Liver Tissue Samples by Inductively Coupled Plasma Mass Spectrometry (ICP-MS) Analysis

Copper, selenium, and zinc contents were determined by ICP-MS in the serum of the 49 HCC patients and in HCC and N tissues of a subset of 27 patients. Tissue sample preparation and mineralization protocol were performed as previously described (19) while serum samples were diluted 1:10 in 0.5**%** HNO_3_. Bovine liver 1577c (National Institute of Standards and Technology, Gaithersburg, MD, USA), Seronorm Trace Elements Serum (SERO AS, Billingstad, Norway), and Clincheck Recipe serum control (Recipe Chemical, Munich, Germany) were used as certified reference material. The ICP-MS analysis was performed as previously reported (19) and the isotopes measured were ^63^Cu, ^66^Zn, ^78^Se, and ^82^Se. ^82^Se was acquired in standard mode while ^63^Cu, ^66^Zn, and ^78^Se were acquired in kinetic energy discrimination (KED) modality with collision cell technology (CCT) system using helium and hydrogen as collision gases. Trace elements concentrations were reported as ng/mg of wet weight for tissues and as µg/L for serum samples.

### Gene Expression Analyses in Liver Tissue Samples

Gene expression analyses of *MT1G*, *MT1H*, and of *SLC39A8* (*ZIP8*), a Zn transporter, were performed by Real-Time RT-PCR in HCC and N tissues of 27 patients. The RNA samples were extracted with TRI**^®^** Reagent following the manufacturer**’**s protocol, treated with TURBO DNA-free**™** Kit (Ambion by Thermo Fisher Scientific, Waltham, MA, USA) and quantified with Qubit**^®^** RNA Broad-Range Assay Kit (Thermo Fisher Scientific). Reverse transcription was obtained using SuperScript**^®^** VILO**™** cDNA Synthesis Kit (Thermo Fisher Scientific) and the gene expression was performed on 7500 Real-Time PCR System using TaqMan**^®^** assays (Applied Biosystem by Thermo Fisher Scientific): *MT1G*, Hs04401199_s1; *MT1H*, Hs00823168_g1; *SLC39A8*, Hs00223357_m1; 18S rRNA, Hs99999901_s1 as endogenous control. The relative gene expression was defined as fold change according to the 2^-ΔΔCt^ method (20).

### Promoter DNA Methylation Analysis by Bisulfite Amplicon Sequencing (BSAS)

Genomic DNA was extracted from HCC and N tissues of 23 patients by Wizard**^®^** Genomic DNA Purification Kit (Promega Corporation, Fitchburg, WI, USA). DNA methylation was assessed by next generation sequencing (NGS) performing BSAS of a region overlapping *MT1G* and *MT1H* promoter. The region of interest was selected among those resulting hypermethylated in HCC tissues, according to our previous microarray-based observations (17). The selected region was located at -598 bp/-999 bp from the transcription start site (TSS) of *MT1G* and it corresponded to the region -1151 bp/-750bp from the TSS of *MT1H*.

#### DNA Bisulfite Conversion and Bisulfite Specific PCR

DNA was quantified using Picogreen (Invitrogen by Thermo Fisher Scientific) and 1 μg of genomic DNA was bisulfite-converted using EZ DNA Methylation according to manufacturer**’**s protocol (Zymo Research, Irvine, CA, USA). Briefly, DNA was bisulfite-converted for 16 h at 50°C and subsequently desulfonated, washed, and eluted in 10 μl of elution buffer. The bisulfite converted region of interest was amplified by the high-fidelity DNA polymerase KOD -Multi & EPi (Toyobo, Osaka, Japan) using specific primers (**Supplementary Table 1**). PCR thermal profile was: 95°C for 4 min; 35 cycles of 95°C for 30 s, 55°C for 30 s, 72°C for 30 s; 72°C for 7 min. PCR products were purified by QIAquick PCR columns (Qiagen, Hilden, Germany), quantified using Picogreen and confirmed through agarose gel electrophoresis.

#### NGS Library Preparation

Library preparation was performed with an Illumina TruSeq Nano DNA library prep kit (Illumina, San Diego, CA, USA) according to the manufacturer**’**s instructions. Briefly, PCR products were end-repaired at 30°C for 30 min and purified by magnetic beads. **“**A**”** nucleotide was added to the 3**’** ends of the blunt fragments using A-tailing mix reagent by incubating at 37°C for 30 min, and then at 70°C for 5 min. Indexing adapters were ligated to the ends of PCR products using ligation mix 2 reagent at 30°C for 10 min. After two washes with sample purification beads, PCR was performed to enrich those DNA fragments carrying adapter molecules on both ends. Thermocycler conditions were as follows: 95°C for 3 min, 8 cycles of 98°C for 20 s, 60°C for 15 s, and 72°C for 30 min, with a final extension at 72°C for 5 min. Quality and length of the libraries were assessed using Agilent 2100 bioanalyzer (Agilent, Santa Clara, CA, USA). Libraries were quantified by qPCR using CFX96 Real Time System (BioRad, Hercules, CA, USA). After normalization, sequencing of the libraries was conducted on the Miseq system (Illumina) with 300 bp paired-end reads.

#### Preprocessing, Genome Mapping, and Methylation Levels Profiling

Potentially existing sequencing adapters and raw quality bases in the low reads were trimmed by Skewer (21). The option -x AGATCGGAAGAGCACACGTCTGAACTCCAGTCA and -y AGATCGGAAGAGCGTCGTGTAGGGAAAGAGTGT were used for the common adapter sequence of the Illumina TruSeq adapters and the option -q 0 -l 25 -k 3 -r 0.1 -d 0.1 was used for trimming low quality 5**’** and 3**’** ends of the raw reads. The cleaned high-quality reads after trimming the low-quality bases and sequencing adapters were mapped to the reference genome by BS-seeker2 software (22). Since the alignment proceeds with reference to some specific genic regions of the genome sequence, 10% miss-mapping rate was allowed. BS-seeker2 was used for the methylation levels profiling at single-base resolution from the mapping results.

### Statistical Analysis

Continuous variables were expressed as mean values ± standard deviations (SD); continuous variables showing a non-Gaussian distribution were log-transformed and expressed as geometric means with 95% confidence intervals (CIs). In particular, for tissue trace elements, Cu and Zn concentrations showed a non-Gaussian distribution so they were log-transformed. Paired Student**’**s t-test was applied to compare pairwise the levels of trace elements and of CGs % methylation between HCC and homologous N tissues. The non-parametric Wilcoxon signed-rank test was applied to compare the expression levels of *MT1G*, *MT1H*, and *ZIP8* between HCC and N tissue: the comparison was performed between the fold change values of each gene and the value of 1, corresponding to the lack of variation in gene expression levels between the two tissues (HCC ***versus*** N tissue). Survival analyses were performed on 47 HCC patients; two subjects were excluded since they deceased during hospitalization due to surgical complications. Kaplan-Meier survival curves were drawn using the log-rank test (Mantel-Cox test) to examine the differences in survival according to serum trace elements. Patients were censored at the last follow-up date up to a maximum of 110 months. Hazard ratio (HR) of mortality with 95% CI was estimated in relation to serum Cu levels by Cox regression analysis adjusted for sex and age, serum Se and Zn concentrations, tumor grading, and microvascular invasion. The Proportional Hazard Assumption was tested by including Time Dependent Covariates in the Cox Model, i.e. a time dependent covariate was generated (by creating interaction between Cu levels and survival time) and included in the Cox model. Kaplan-Meier survival curves were drawn also according to the methylation levels of specific CGs located in the promoter region of *MT1G/MT1H*. The median methylation level of CG4, CG5 and CG6 was considered. All the analyses were performed using the IBM SPSS 20 statistical software (IBM Inc, Armonk, NY, USA) and a P-value **<**0.05 was considered statistically significant.

## Results

### Clinical and Biochemical Characteristics of HCC Patients

Forty-nine HCC patients, 42 males and 7 females, were enrolled for this study. Their main clinical and biochemical characteristics are reported in **Table 1**. They had a mean age of 70.8 ± 6.9 years and were all free from hepatitis B or C infections, according to the enrolment criteria. Biochemical indexes of hepatic function were consistent with a compensated liver disease, while the mean alpha-fetoprotein levels (24.8 µg/L) were above the reference value of 7 µg/L. All patients were also evaluated for serum levels of Cu, Se, and Zn and the mean values attested within the normal range according with the data of the Italian “Istituto Superiore di Sanità,” in agreement with the classification of the World Health organization (23, 24) (**Supplementary Figure 1**). Analyses were also performed for possible correlation among serum concentrations of Cu, Zn, and Se (**Supplementary Table 2**) and for correlation among the serum trace elements and clinical variables (**Supplementary Table 3**).

**Table 1:** Clinical and biochemical characteristics of HCC patients.

Parameters	Reference values****	HCC patients (n = 49)****
Age (years)		70.8 ± 6.9
Gender (male/female)		42/7
Tumor grading* (%):		
1		25.0
2		67.5
3		7.5
Microvascular invasion* (%)		40.0
Hemoglobin (g/dl)	13.5–16	13.5 ± 1.85
Hematocrit (%)	38.0–49.0	41.5 ± 5.16
MCV (fl)	86.0–98.0	91.5 ± 8.43
Platelets (10^9^/L)^a^	150–400	205 (181–233)
White blood cells (10^9^/L)^a^	4.30–10.00	6.45 (5.78–7.19)
CRP (mg/L)^a^	<5.00	6.38 (4.36–9.34)
ESR (mm/h)	<37.0	38.7 ± 30.4
Albumin (g/L)	35.0–50.0	39.6 ± 5.8
AST (U/L)^a^ ^a^	5.0–50.0	41.7 (33.2–52.2)
ALT (U/L)^a^	6.0–50.0	41.5 (32.2–53.5)
ALP (U/L)^a^	50.0–130.0	88.9 (77.5–101.8)
CHE (U/L)	5,000–17,000	6574 ± 1776
GGT (U/L)^a^	4.0–60.0	77.2 (60.4–98.7)
Total bilirubin (mg/dl)^a^	0.11–1.05	0.79 (0.68–0.91)
Direct bilirubin (mg/dl)^a^	<0.35	0.28 (0.24–0.32)
IgA (g/L)^a^	0.70–4.00	3.03 (2.38–3.86)
Alpha-fetoprotein (µg/L)^a^ ^a^	<7.0	24.8 (12.1–51.0)
Serum trace elements^b^:		
Cu (µg/L)	648–1,301	934 ± 243
Se (µg/L)	63–160	80.4 ± 16.4
Zn (µg/L)	597–1,028	651 ± 117

*tumor grading and microvascular invasion data were available for 40 out of 49 subjects (81.6%).

a log-transformed variables are shown as geometric mean with 95% confidence interval

b for reference values for serum trace elements see Refs. 23, 24.

MCV, mean corpuscular volume; CRP, C-reactive protein; ESR, erythrocyte sedimentation rate; AST, Aspartate aminotransferase; ALT, Alanine aminotransferase; ALP, Alkaline phosphatase; CHE, Cholinesterase; GGT, Gamma-glutamyltranspeptidase.

### Survival Rate According to Serum Trace Elements Content

**Figure 1:**
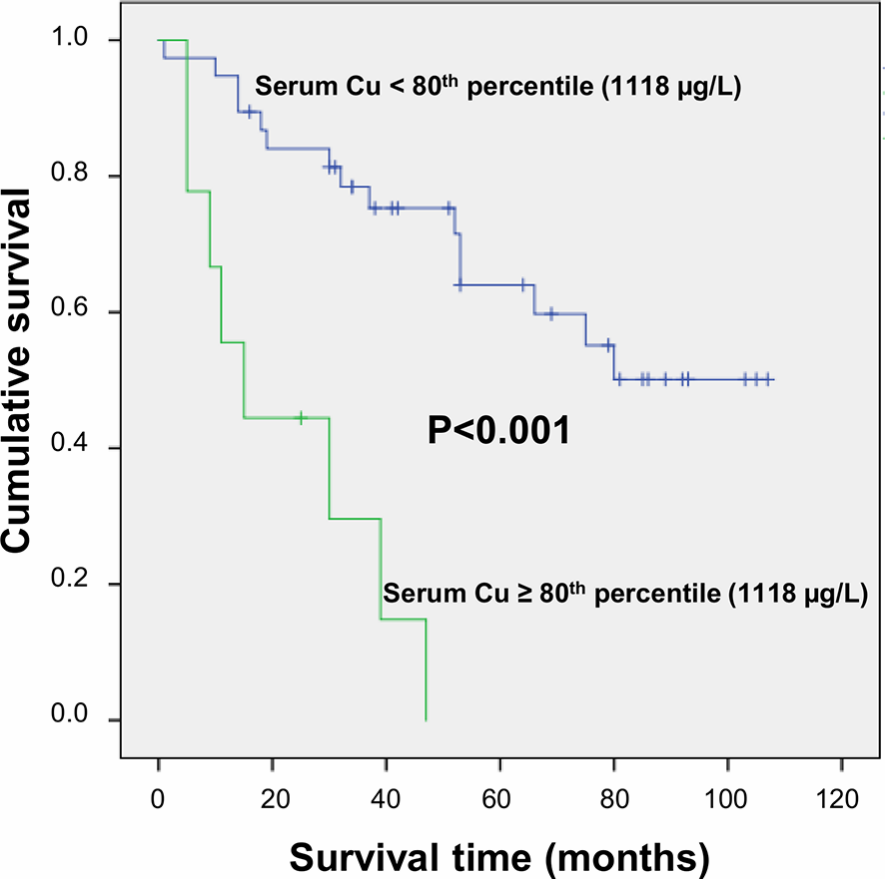
Survival curves plotted by Kaplan-Meier analysis according to serum Cu levels. HCC patients with serum Cu concentration above the 80^th^ percentile (1,118 µg/L) showed significantly increased mortality as compared to patients with serum Cu concentration <1,118 µg/L. Patients were censored at the last follow-up date.

After a follow-up period of a median time of 40 months, 23 deaths (48.9%) were observed. Serum trace elements concentrations were analyzed according to patient’s survival rate. By univariate Cox regression analysis, serum Cu levels were directly associated to increasing mortality (P = 0.009), while no significant association was found for Se and Zn serum levels (P = 0.136 and P = 0.256, respectively). This result was confirmed after adjustment for sex and age (P = 0.001), as well as by including all the trace elements as dependent variables, with only Cu levels maintaining a significant association with mortality (P = 0.002). The association between serum Cu levels and mortality was confirmed even after adjustment for tumor grading and microvascular invasion (P = 0.001). A time dependent covariate of serum Cu levels was included in the Cox model and was not significant (P = 0.483), thereby indicating that proportional hazard assumption was not violated. Stratifying the study population according to quintiles of serum Cu concentrations, Kaplan-Meier survival curves showed that mortality rate increased by increasing Cu levels, with a very high rate (88.9%) among subjects within the highest quintile of distribution (P = 0.025 by Log Rank test for trend). Considering a threshold value at the 80^th^ percentile of Cu levels (1,118 µg/L), subjects with Cu concentration above this value had a significantly decreased survival rate (P < 0.001 by Log-Rank test—**Figure 1**) with a marked increased hazard ratio for mortality (HR 6.88 with 95% CI 2.60–18.23, P < 0.001 by Cox regression). High Cu serum concentrations remained associated with an increased risk of mortality after adjustment for sex and age, Zn and Se levels, and even tumor grading and microvascular invasion in the regression models (**Table 2**). The significance was maintained also in the adjusted model in which all the variable showing a correlation with serum Cu levels were included, i.e. hemoglobin, hematocrit, platelets, white blood cells, CRP, ESR, albumin, ALP, and GGT (**Table 2**).

**Table 2:** Hazard Ratios (HR) associated to 80^th^ percentile of Cu levels calculated by Cox regression analysis.

	HR (95% CI)	P value
Univariate	6.88 (2.60–18.23)	<0.001
Sex and age adjusted	7.70 (2.75–21.58)	<0.001
Sex, age, serum Se, serum Zn adjusted	7.58 (2.63–21.81)	<0.001
Tumor grading and microvascular invasion adjusted^*^	5.66 (1.77–18.2)	0.004
Biochemical data adjusted^#^	11.65 (2.26–60.04)	0.003

*data available for 40 out of 49 subjects (81.6%).

^#^Hemoglobin, Hematocrit, Platelets, White blood cells, CRP, ESR, Albumin, ALP, GGT adjusted.

### Trace Elements Quantification in HCC and Homologous Non-Neoplastic Liver Tissue

**Figure 2:**
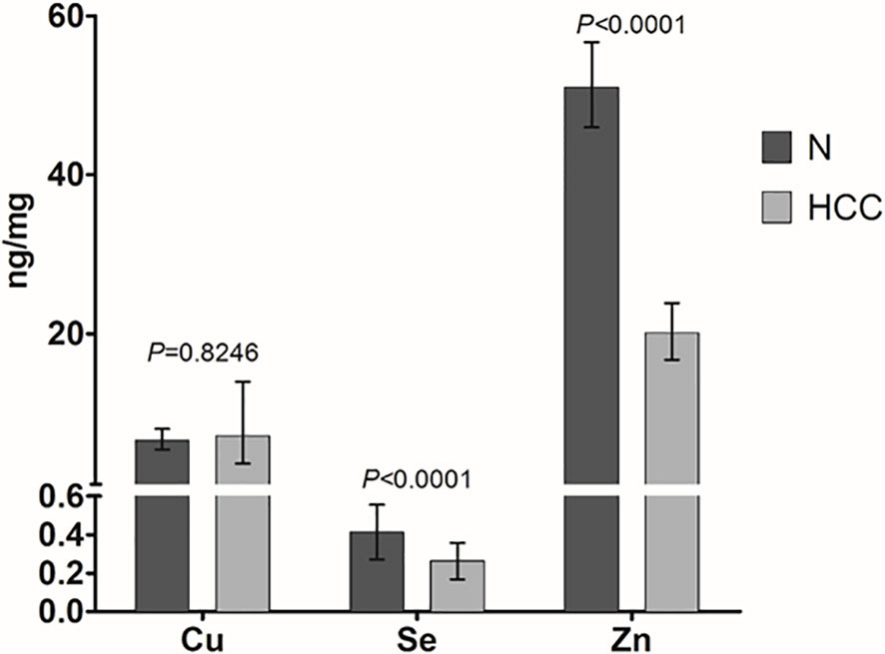
Mean trace elements content in HCC and non-neoplastic liver (N) tissue. Tissue Cu and Zn concentrations are expressed as geometric mean with 95% confidence interval while Se concentrations are expressed as mean ± SD. Paired Student’s t-test was applied.

**Figure 3:**
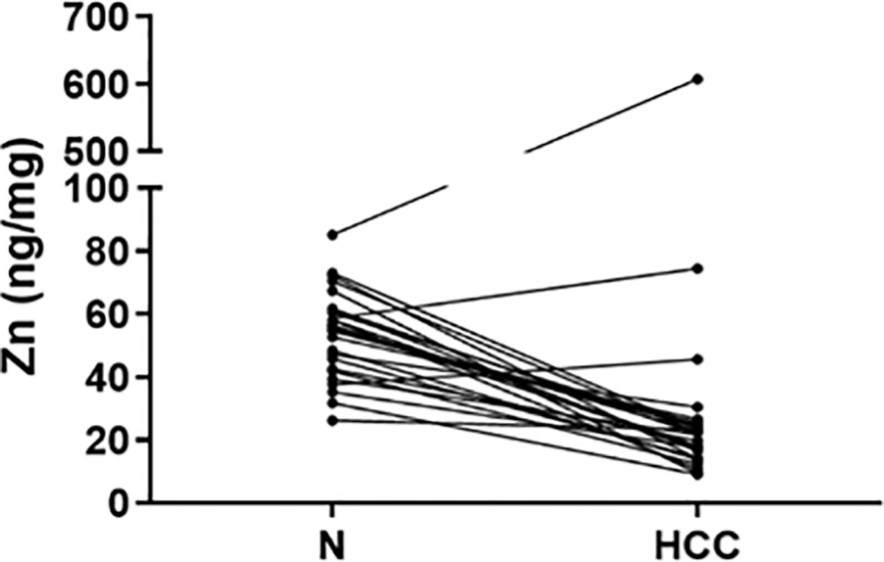
Zinc tissue content in HCC and homologous non-neoplastic liver (N) tissues. Zn levels were reduced in the majority of HCC tissues while 2 HCC tissues showed extremely high Zn levels.

Cu, Se, and Zn content was determined by ICP-MS in liver tissues, i.e. HCC and N tissues, of a subset of 27 patients (23 males and 4 females with a mean age of 70.6 ± 6.7 years). As shown in **Figure 2**, Se and Zn concentrations were lower in HCC tissues as compared to N tissues and the differences were statistically significant (P < 0.0001). On the contrary, mean Cu content did not show statistically significant differences for the comparison of HCC with N tissues. Looking more specifically at each single subject data, Se levels were reduced in the great majority of HCC tissues, i.e. 20 out of 27. Similarly, Zn levels were reduced in 24 out of 27 HCC tissues while 2 HCC tissues showed extremely high Zn levels that paralleled with the increase in MT1G and MT1H gene expression (**Figure 3**). As for Cu content, the tendency was not so evident, and the differences were not statistically significant. In particular, 13 patients showed an increase of Cu levels in HCC tissue as compared to N tissue.

### *MT1G*, *MT1H*, and *ZIP8* Gene Expression Analysis in Liver Tissues

**Figure 4:**
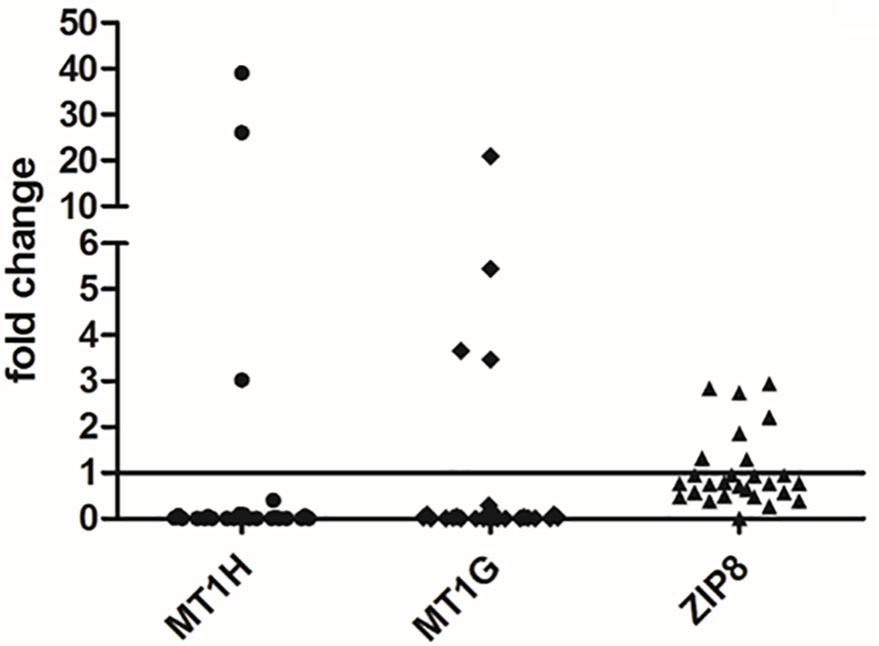
Gene expression analysis of *MT1G*, *MT1H*, and *ZIP8* in liver tissues. The differences in the mRNA levels in HCC as compared to non-neoplastic liver tissue (N) are expressed as fold change according to the 2^-ΔΔCT^ method. Wilcoxon test was applied to compare pair-wise the mRNA levels between HCC and N tissues.

Gene expression analysis was performed for the two MTs, i.e. *MT1G* and *MT1H*, able to bind divalent heavy metal ions such as Cu and Zn and for a zinc transporter, i.e. *SLC39A8* (also known as *ZIP8*). As shown in **Figure 4**, both *MT1G* and *MT1H* were strongly repressed in the majority of HCC tissues. Precisely, *MT1H* was repressed in 24 out of 27 HCC tissue samples and *MT1G* was repressed in 23 out of 27 HCC tissues. The two HCC tissues showing a marked increase in *MT1G* and *MT1H* expression were those with very high Zn content (**Figure 3**). On the contrary, the Zn transporter *ZIP8* did not show a statistically significant difference for transcriptional regulation in HCC tissues as compared to N tissues (P = 0.313).

### Promoter DNA Methylation of *MT1G* and *MT1H* According to Survival Rate

**Figure 5:**
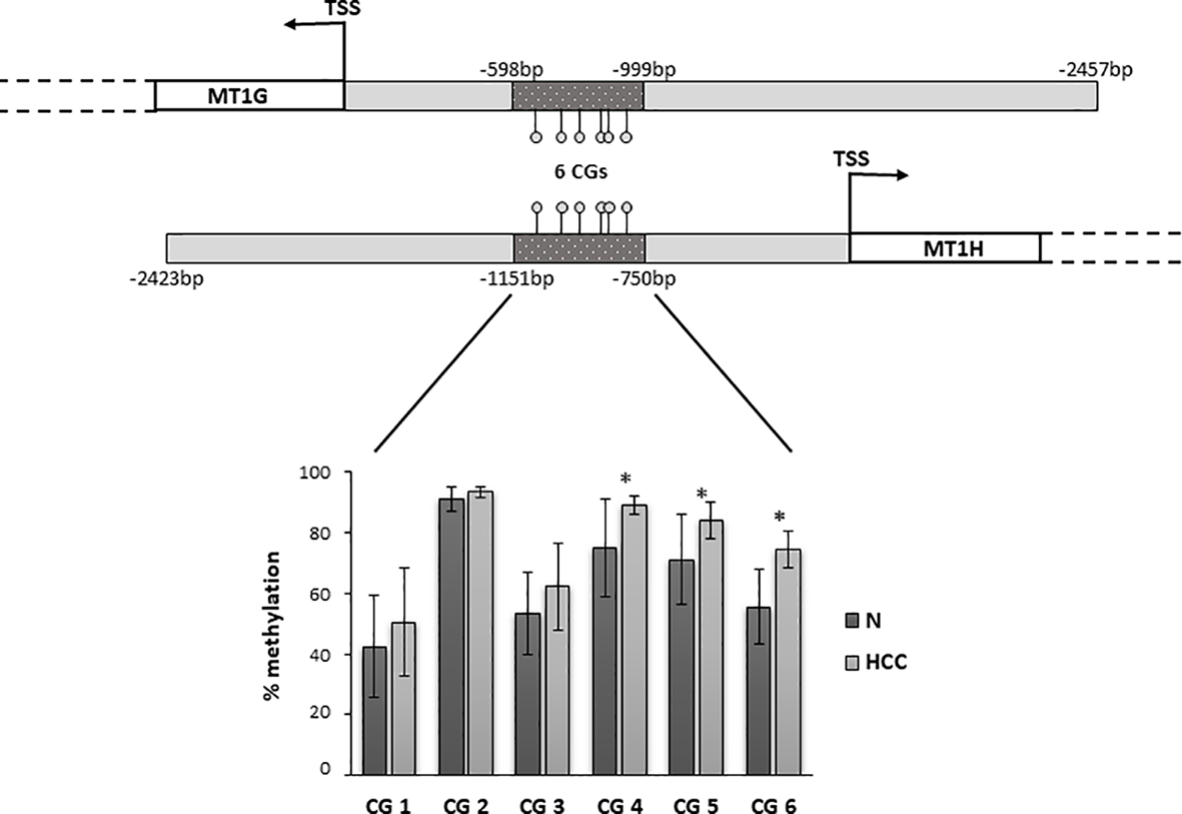
Promoter region of *MT1G* and *MT1H* genes with the localization of the region analyzed by bisulfite amplicon sequencing (BSAS). Percentage of DNA methylation of specific CpGs in the promoter region of MT1G and MT1H. CG4, CG5, and CG6 were significantly hypermethylated in hepatocellular carcinoma (HCC) as compared to non-neoplastic liver (N) tissue (*P < 0.05). TSS, transcription start site.

**Figure 6:**
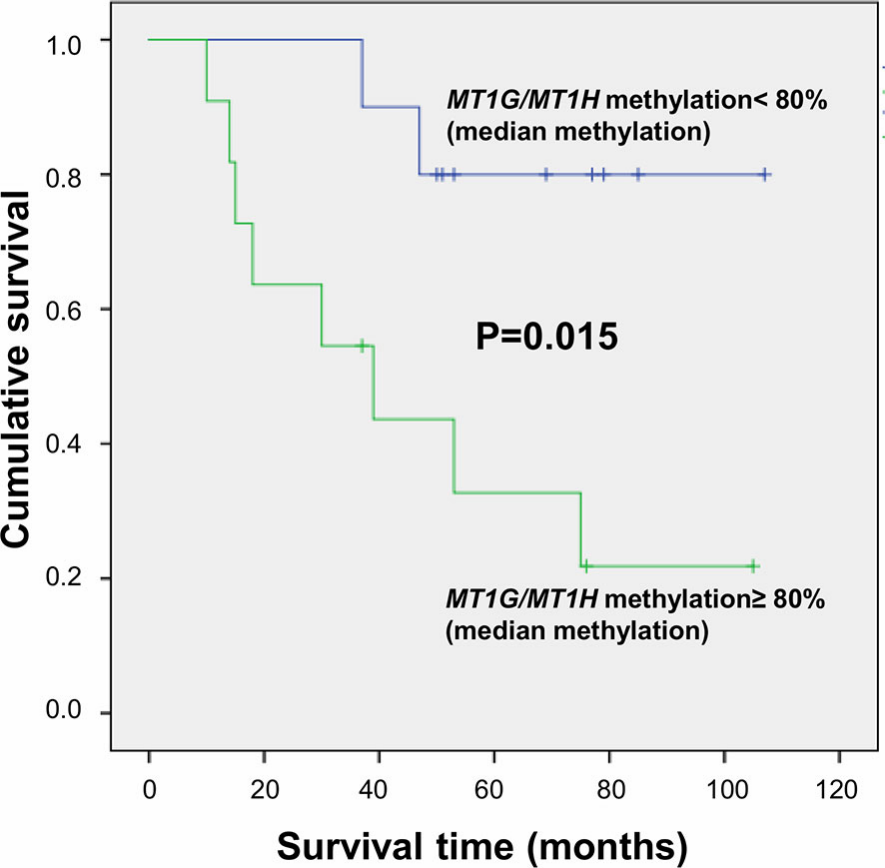
Survival curves plotted by Kaplan-Meier analysis according to median percentage of methylation of CG4, CG5, and CG6 in HCC tissues. Higher MT1G/MT1H promoter methylation levels are associated to increased mortality.

Metallothioneins promoter DNA methylation was analyzed by BSAS in HCC and N tissues of 23 patients. **Figure 5** shows a scheme of the *MT1G* and *MT1H* gene promoter regions. The overlapping region between the two promoters that contains the six CGs sites analyzed by BSAS is highlighted. Nine out of 19 HCC tissues showing MTs down-regulation presented at least one hypermethylated CpG among the six CpGs analyzed by BSAS. Three CpG sites, precisely CG4, CG5, and CG6, were significantly hypermethylated in HCC tissue as compared to N tissue (P < 0.05). The methylation levels of the three CGs that resulted hypermethylated in HCC tissues, were then analyzed in relation to survival rate. Considering the median methylation levels of the three hypermethylated CGs in HCC (80% methylation), patients with higher methylation values showed increased mortality rate (P = 0.015, **Figure 6**).

## Discussion

The contribution of trace elements in carcinogenesis recently gained increasing interest both for the identification of new pathogenetic mechanisms (4, 25–27) and the identification of prognostic factors (28). In this study, we precisely investigated the role of trace elements in liver carcinogenesis and the possible association with HCC survival rate. We also evaluated the expression levels of specific MTs, proteins involved in trace elements homeostasis, their epigenetic regulation through promoter DNA methylation, and the possible connection with HCC prognosis. Through this approach we aimed at approaching the role of trace elements concentrations related both to clinical outcomes, as a possible prognostic marker of disease in HCC, and to epigenetic mechanisms regulating metallothioneins gene expression. The patients enrolled in this study were carefully selected to be free from HCV/HBV infection, in order focus mainly on factors different from those commonly known to be associated to liver cancer development. Serum trace elements levels were within the normal range (23), even if Se and Zn concentrations were very close to the lowest reference values, consistently with previous findings (5). Interestingly, serum Cu levels were directly associated with a significantly decreased survival rate and those patients with serum Cu levels in the highest quintile had a mortality risk more than six-fold higher as compared to patients within the other four quintiles groups, substantially higher HR than previously reported in a cohort of Chinese patients (28). That study had also reported an association of Cu/Zn ratio with increased mortality, while results from the present study show that the statistical significance is probably driven by the effects of serum Cu levels. As previously suggested by Balter and colleagues, the effects on serum Cu levels in HCC patients could depend on the reallocation in the body of the fraction of Cu bound to cysteine-rich proteins such as MTs (29). In order to get valuable insights in the relationship between trace elements and HCC, we also quantified trace elements content in the hepatic tissues, and we also assessed MTs expression levels in the liver. Se and Zn contents were significantly reduced in HCC tissues as compared to N tissues and these data support previous observations (4). As for Se, our data confirm the reduced Se content in HCC tissue as previously reported by two studies (30, 31) whose data remained controversial since the differences were not, in all cases, statistically significant (30). A reduced amount of Zn in cancer tissue was observed by several studies and seems to be a specificity of HCC (27). In our study we observed the reduction of Zn content in HCC tissues and the concomitant down-regulation of MTs, precisely of *MT1G* and *MT1H*. Surprisingly, in 2 out of 27 HCC tissues, Zn content was extremely high and the two MTs were up-regulated. These data confirm the strong link between Zn levels and MTs regulation and suggest the need for further investigations on the role of Zn in HCC. In fact, intracellular Zn has a dual role, namely an anti-oxidant function but, in case of overload, it can also promote cellular oxidative stress (9). As for Cu content, it was highly variable in the HCC tissues collected in the present study, and the differences observed for the comparison with non-neoplastic tissue were not statistically significant. Hence, the relationship between Cu content and MTs expression does not appear as univocal as that with Zn, and leads toward the hypothesis of the involvement of other possible regulatory mechanisms, namely other MTs isoforms or other proteins able to bind divalent metals, hypotheses to be explored by *ad hoc in vitro* studies Reports form the literature are not consistent in this regard, with studies describing an increased Cu content (6, 30, 32, 33) and others reporting a decreased Cu content in HCC (34). The hypothesis of promoter DNA methylation as a possible epigenetic mechanism responsible for *MT1G* and *MT1H* down-regulation in HCC came from our previous observations by genome-wide microarray-based approach (17) and from other studies that observed a promoter DNA hypermethylation of *MT1G* in HCC (11, 35, 36). In the present study, we propose that the hypermethylation of a specific region is involved in the simultaneous repression of both *MT1G* and *MT1H*. The finding of *MT1H* as a possible tumor suppressor gene silenced by promoter hypermethylation is novel for HCC and previously observed only in prostate cancer (37). Another interesting result refers to the association of higher *MT1G*/*MT1H* promoter methylation levels with increased mortality. Previous observations in this regard are limited, conflicting, and sometimes without statistical significance. Recently, Zeng and colleagues observed an unexpected association of *MT1G* hypermethylation with good survival (35) while Kanda and colleagues previously observed a worse survival associated with *MT1G* hypermethylation but without reaching statistical significance (36). Findings from the present study could help to clarify this issue and, furthermore, suggest a new possible epigenetic prognostic factor for HCC identified at early stages. The findings from the present study are also helpful for a better understanding of the link between trace elements and HCC by suggesting the possible role of serum Cu concentrations and of *MT1G*/*MT1H* promoter methylation in cancer tissues as prognostic factors in HCC. This study has some limitations among which the relatively small sample size of the group of cancer patients evaluated, and the missing data for some parameters of the enrolled patients, both of which may have limited reaching the statistical significance for certain outcomes. Although further studies specifically focused on unveiling the features underlying such relationships at a cellular and molecular level are needed, the present observations highlight a novel perspective for a potential etiopathogenetic and preventive approach by addressing epigenetic mechanisms of disease.

## Data Availability Statement

The datasets presented in this study can be found in online repositories. The names of the repository/repositories and accession number(s) can be found below: NCBI SRA (Accession: PRJNA662561).

## Ethics Statement

The studies involving human participants were reviewed and approved by Ethical Review Board of the University of Verona School of Medicine Hospital (Verona, Italy). The patients/participants provided their written informed consent to participate in this study.

## Author Contributions

SU and DS performed analysis and interpretation of data, and wrote the manuscript. SF conceived study design, performed analysis and interpretation of data, and wrote the manuscript. FM, SM, AR, AC, PP, GB, AF, NM, and FA contributed significantly to data collection, analysis and interpretation, and critical revision. AG, FP, OO, and S-WC contributed significantly to interpretation of data and revising the intellectual content. All authors contributed to the article and approved the submitted version.

## Conflict of Interest

The authors declare that the research was conducted in the absence of any commercial or financial relationships that could be construed as a potential conflict of interest.
