# Supplementary material for: Trace Elements Status and Metallothioneins DNA Methylation Influence Human Hepatocellular Carcinoma Survival Rate
Source: Front Oncol. 2021 Jan 28;10:596040. doi: 10.3389/fonc.2020.596040 (PMC7876470; doi:10.3389/fonc.2020.596040)
Supplement: Supplementary file 1 [file DataSheet_1.docx]

Supplementary Material

# Supplementary Tables

| **Supplementary Table 1: Primer sequences specific for MT1G/MT1H promoter region bisulfite-converted** | |
| --- | --- |
| **Primer** | **Sequence** |
| Forward | 5’-TTTTGAGGAGTTGAATGGGTTTA-3’ |
| Reverse | 5’- AAATTCACACCAAACAATACCACTT -3’ |

| **Supplementary Table 2: Correlation analyses among serum trace elements** | | | | | |  |
| --- | --- | --- | --- | --- | --- | --- |
|  | **serum Cu (µg/L)** | | **serum Zn (µg/L)** | | **serum Se (µg/L)** | |
|  | **r** | **P value** | **r** | **P value** | **r** | **P value** |
| **serum Cu (µg/L)** | 1 |  | -0.188 | 0.206 | -0.106 | 0.480 |
| **serum Zn (µg/L)** | -0.188 | 0.206 | 1 |  | 0.327^*^ | 0.025 |
| **serum Se (µg/L)** | -0.106 | 0.480 | 0.327^*^ | 0.025 | 1 |  |
| **r: Pearson correlation coefficient** | | |  |  |  |  |
| ***P value ≤ 0.05** |  |  |  |  |  |  |

| **Supplementary Table 3: Correlation analyses between trace elements and clinical variables** | | | | | | |
| --- | --- | --- | --- | --- | --- | --- |
|  | **serum Cu (µg/L)** | | **serum Zn (µg/L)** | | **serum Se (µg/L)** | |
|  | **r** | **P value** | **r** | **P value** | **r** | **P value** |
| Age (years) | 0.032 | 0.830 | 0.050 | 0.736 | 0.070 | 0.642 |
| Hemoglobin (g/dL) | -0.535^**^ | <0.001 | 0.475^**^ | 0.001 | 0.337^*^ | 0.02 |
| Hematocrit (%) | -0.495^**^ | <0.001 | 0.491^**^ | <0.001 | 0.299^*^ | 0.041 |
| MCV (fL) | -0.141 | 0.346 | -0.054 | 0.718 | -0.097 | 0.516 |
| Platelets (10^9^/L) | 0.418^*^ | 0.003 | 0.016 | 0.916 | 0.230 | 0.120 |
| White blood cells (10^9^/L) | 0.299^*^ | 0.041 | 0.299^*^ | 0.041 | 0.126 | 0.398 |
| CRP (mg/L) | 0.567^**^ | <0.001 | -0.057 | 0.703 | -0.052 | 0.726 |
| ESR (mm/h) | 0.679^**^ | <0.001 | -0.404^*^ | 0.005 | -0.178 | 0.237 |
| Albumin (g/L) | -0.493^**^ | <0.001 | 0.431^*^ | 0.003 | 0.300^*^ | 0.040 |
| AST (U/L) | 0.161 | 0.291 | 0.074 | 0.631 | -0.088 | 0.566 |
| ALT (U/L) | 0.062 | 0.677 | 0.125 | 0.402 | 0.033 | 0.827 |
| ALP (U/L) | 0.405^*^ | 0.005 | -0.250 | 0.094 | -0.144 | 0.339 |
| CHE (U/L) | -0.233 | 0.133 | 0.403^*^ | 0.007 | 0.263 | 0.089 |
| GGT (U/L) | 0.466^**^ | 0.001 | -0.106 | 0.482 | -0.105 | 0.485 |
| Total bilirubin (mg/dL) | 0.041 | 0.783 | -0.149 | 0.319 | -0.066 | 0.660 |
| Direct bilirubin (mg/dL) | 0.065 | 0.676 | -0.210 | 0.170 | -0.213 | 0.166 |
| IgA (g/L) | 0.115 | 0.475 | -0.271 | 0.087 | -0.173 | 0.281 |
| Alpha-fetoprotein (µg/L) | 0.263 | 0.080 | 0.068 | 0.656 | -0.002 | 0.990 |
| **r: Pearson correlation coefficient** | |  |  |  |  |  |
| ***P value ≤ 0.05** |  |  |  |  |  |  |
| ****P value ≤ 0.001** |  |  |  |  |  |  |
| Abbreviations: MCV, mean corpuscular volume; CRP, C-reactive protein; ESR, erythrocyte sedimentation rate; AST, Aspartate aminotransferase; ALT, Alanine aminotransferase; ALP, Alkaline phosphatase; CHE, Cholinesterase; GGT, Gamma-glutamyltranspeptidase. | | | | | | |

# Supplementary Figure


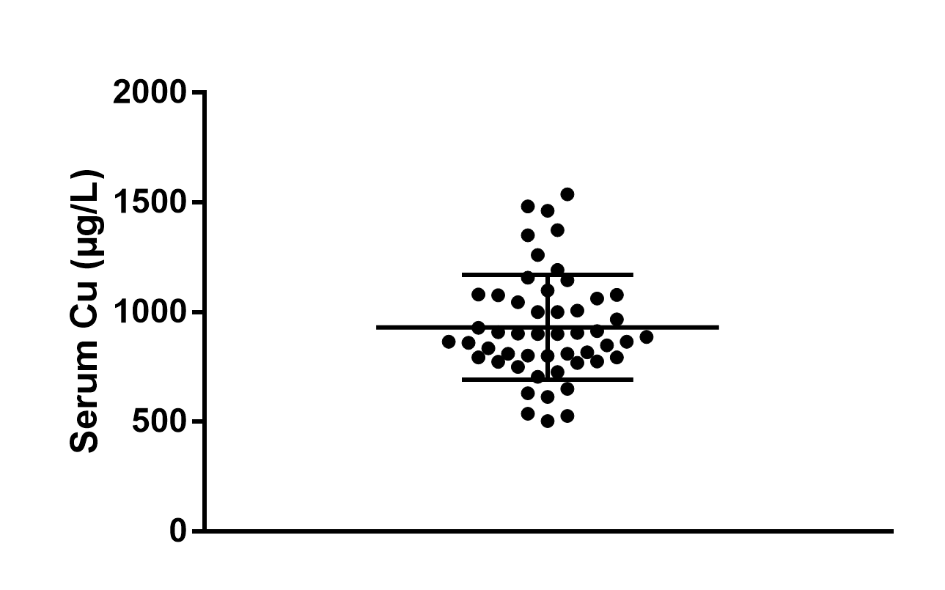


**Supplementary Figure 1.** Scatter plot of serum Cu concentrations of HCC patients.
